# Association of C-reactive protein with all-cause and cause-specific mortality in people with gout

**DOI:** 10.1186/s40001-024-01923-3

**Published:** 2024-06-10

**Authors:** Lishuai Han, Lijuan Zhang, Wenlu Hu, Yang Lu, Zhenwei Wang

**Affiliations:** 1 Department of Rheumatology and Immunology, The First Affiliated Hospital of Zhengzhou University, Zhengzhou, 450052 China; 2 Department of Cardiology, The First Affiliated Hospital of Zhengzhou University, Zhengzhou, 450052 China

**Keywords:** C-reactive protein, All-cause mortality, Cardiovascular mortality, Cancer mortality, NHANES

## Abstract

**Aims:**

To test the association of C-reactive protein (CRP) with all-cause and cause-specific mortality in people with gout.

**Methods:**

This cohort study included 502 participants with gout from the National Health and Nutrition Examination Survey. Multivariate Cox regression analysis, subgroup analysis, and restricted cubic spline (RCS) analyses were utilized to examine the association of CRP levels with all-cause, cardiovascular, and cancer mortality.

**Results:**

After adjusting for multiple variables, Cox regression analysis showed that compared with individuals in the lowest tertile of CRP levels, those in the middle and highest tertiles experienced increases in all-cause mortality risk of 74.2% and 149.7%, respectively. Similarly, the cancer mortality risk for individuals in the highest tertile of CRP levels increased by 283.9%. In addition, for each standard deviation increase in CRP, the risks of all-cause and cancer mortality increased by 25.9% and 35.4%, respectively (*P* < 0.05). Subgroup analyses demonstrated that the association between CRP levels and all-cause mortality remained significant across subgroups of age (≤ 60 and > 60 years), gender (male), presence or absence of hypertension, non-diabetes, cardiovascular disease, non-cardiovascular disease and non-cancer. Furthermore, the association with cancer mortality was significant in subgroups including males, those without hypertension and cancer, and those with or without diabetes. However, the association with cardiovascular mortality was only significant in the non-hypertension subgroup (*P* < 0.05). Nonlinear association of CRP with all-cause mortality and linear association with cancer mortality were also confirmed (*P* for nonlinearity = 0.008 and 0.135, respectively).

**Conclusions:**

CRP levels were associated with increased all-cause and cancer mortality among individuals with gout.

## Introduction

Gout has become an important public health issue alongside socio-economic advancements. The Global Burden of Disease study has reported that from 1990 to 2019, the total number, age-standardized prevalence and incidence of gout patients have shown a continuous upward trend, increasing alongside socio-demographic indices. Regions such as Australasia, North America, and China experience a higher disease burden from gout than the global average [1–5]. Moreover, the global prevalence of gout has substantially increased within the younger age group of 15–39 years over the past 30 years [6]. In addition, the impact of gout on all-cause and cardiovascular mortality has been demonstrated [7, 8]. These data suggest that early intervention for gout patients is imperative to reduce the disease’s burden. Current studies indicate that hypertension, high alcohol consumption, high body mass index (BMI), and renal insufficiency are major risk factors for gout, while inflammation may also play a role in the development of gout and its complications [9–12].

In clinical practice, C-reactive protein (CRP), synthesized by the liver, is one of the major acute-phase proteins reflecting the state of inflammation. Its blood levels change rapidly in response to inflammatory reactions and tissue damage [13]. However, current evidence suggests that CRP is not only a biomarker for various acute and chronic inflammatory conditions but also has been implicated in cardiovascular disease (CVD) and metabolism-related disorders, such as gout [14–17]. In addition, the association between CRP and mortality has garnered significant attention due to its role as an inflammatory biomarker, reflecting the state of inflammation within the body [18–20]. Inflammation is considered a key factor in the development of various chronic diseases, including CVD, diabetes, and certain types of cancer. Moreover, gout, a chronic metabolic disease with globally rising prevalence, often presents with elevated CRP levels, suggesting a possible link to systemic inflammation [17]. Patients with gout face a higher risk of both all-cause and cause-specific mortality, due to their unique metabolic abnormalities and chronic inflammatory responses, compared to the general population [7, 8]. However, current research on the association between CRP levels and mortality risk in gout patients is relatively scarce, especially lacking in-depth exploration of the link between CRP and specific causes of death. Therefore, studying the relationship between CRP and both all-cause and cause-specific mortality in the gout population can help unveil the potential mechanisms by which gout impacts individual health, particularly in assessing mortality risk and developing personalized management strategies for gout patients.

Investigating the connection between CRP and mortality rates in gout patients is essential as it provides valuable insights into how controlling inflammation can optimize the long-term health and survival rates of these patients. Understanding the relationship between CRP and mortality risk in gout patients not only aids in enhancing our current understanding of the impact of gout but may also guide the management of inflammation in clinical practice, thereby reducing mortality risks in gout patients. Through such research, we can better identify high-risk gout patients and provide them with targeted interventions to improve their prognosis. Therefore, this study proposed to investigate the correlation between CRP levels and both all-cause and cause-specific mortality in gout participants from the National Health and Nutrition Examination Survey (NHANES).

## Methods

### Study participants

**Fig. 1:**
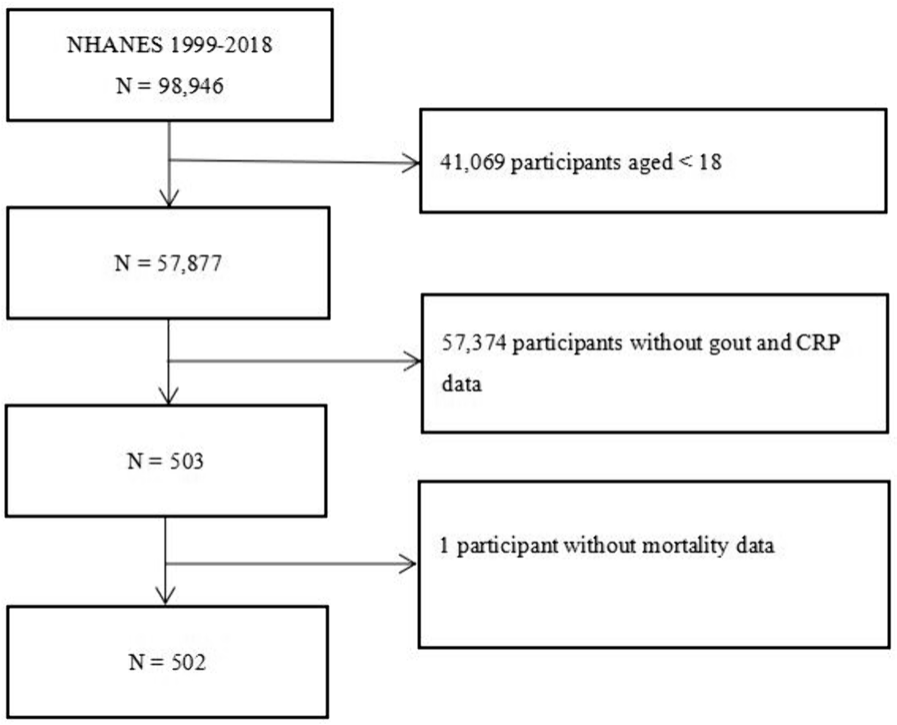
Flowchart of the study population. NHANES, National Health and Nutrition Examination Survey; CRP, C-reactive protein

NHANES is a national survey conducted by the National Center for Health Statistics (NCHS) of the Centers for Disease Control and Prevention (CDC). Its purpose is to monitor the health and nutritional status of adults and children across the U.S. and to provide vital health statistics for the nation. It has been conducted biennially since 1990. Specific study design, content, and details about NHANES are available on its publicly accessible website (https://www.cdc.gov/nchs/nhanes/). In this large cohort study, a total of 502 individuals from the NHANES 2007–2010 cycle were enrolled (Fig. 1). Inclusion criteria were: (1) patients diagnosed with gout; (2) age 18 and above; and (3) provided complete baseline data at the start of the study, including CRP levels, vital signs, laboratory results, and lifestyle information. Exclusion criteria: (1) lack of complete data on CRP levels or baseline information; (2) presence of serious comorbidities at the start of the study, such as end-stage renal disease; (3) history of using immunosuppressants or biologics in the year prior to the study start; and (4) inability to provide mortality data during follow-up. Process for participant identification and data extraction: Utilizing the NHANES database, individuals meeting the inclusion criteria were filtered through predefined query conditions. A further review was conducted on the initially filtered individuals to ensure all inclusion criteria were met and all exclusion criteria were addressed. For those who qualified, an automated data extraction tool was used to retrieve complete baseline data and mortality data during follow-up from the database. Data extraction included personal biomarker data (such as CRP levels), clinical data, lifestyle information, and mortality records during follow-up. The NHANES protocol complied with the basic principles of the Declaration of Helsinki and was approved by the NCHS of the CDC Institutional Review Board. All participants signed an informed consent form for their participation in NHANES.

### Covariates collection and definitions

This study included covariates such as demographic variables, anthropometric measurements, comorbidity factors, medication use, and biomarkers. Ideal exercise was defined as engaging in at least 75 min of vigorous activity or 150 min of moderate-intensity exercise weekly [21]. Smoking status was categorized into three groups based on frequency: every day, some days, and not at all. Drinking was defined as consuming at least 12 glasses of alcohol in the past year. Hypertension was identified either by a previous diagnosis from a doctor or by a current systolic blood pressure (SBP)/diastolic blood pressure (DBP) of ≥ 140/90 mmHg [22]. Diabetes was determined by a prior diagnosis or a current fasting plasma glucose (FPG) ≥ 7.0 mmol/L or hemoglobin A1c (HbA1c) ≥ 6.5% [23]. Hypercholesterolemia, CVD, and cancer assessments were based on the health status information provided by participants during NHANES family interviews, where CVD encompassed heart failure, angina, heart attack, and stroke. Information on the use of hypotensive drugs, hypoglycemic drugs and cholesterol-lowering drugs was collected through the family interview questionnaires. Trained investigators measured height, weight and blood pressure of participants according to standard procedures, calculating BMI as weight (kg) divided by height in meters squared (m^2^). Blood markers were collected by professionals and analyzed in a standard laboratory, with CRP levels quantitatively determined by latex-enhanced turbidimetry and interpreted using a calibration curve by laboratory professionals at the University of Washington.

### Determination of mortality

Mortality information was obtained from the NHANES public-use linked mortality file, which was matched to NHANES participants. This included all-cause mortality, cardiovascular mortality, and cancer mortality as determined by ICD-10 codes. For this study, all participants were followed from the time of their participation in the NHANES interview until either their death or December 31, 2015.

### Statistical analysis

Similar to previous studies [18, 20], this research aimed to explore the potential nonlinear relationship between CRP levels and the risk of mortality, as well as to capture the gradient effect of CRP levels on mortality risk more effectively. Therefore, participants were categorized into three groups based on the tertiles of CRP: T1: ≤ 0.16 mg/dL, T2: 0.16–0.45 mg/dL, T3: > 0.45 mg/dL. Differences in continuous variables among the three groups were assessed using one-way ANOVA or non-parametric tests, with continuous variables expressed as mean ± standard deviation or median (1st quartile, 3rd quartile). Differences in categorical data among the three groups were assessed using the Chi-square test, with categorical variables expressed as frequencies and percentages. Three models were created for multivariate Cox regression analysis based on the results of univariate Cox regression analysis: model 1 was unadjusted, model 2 was adjusted for age and sex, and model 3 was adjusted for age, sex, and covariates that were *P* < 0.05 in univariate Cox regression analysis. In addition, CRP was standardized to eliminate differences in units of measure, to improve model stability and to facilitate interpretation; that is, the standardized CRP value = (original value of CRP−mean value)/standard deviation. Kaplan–Meier survival analysis was used to assess differences in survival probabilities for all-cause, cardiovascular, and cancer mortality among the three CRP groups over the follow-up time. Subgroup analyses based on age, sex, hypertension, diabetes, CVD, and cancer were used to examine stratified associations between CRP and mortality. In addition, restricted cubic spline (RCS) plots were used to explore the potential nonlinearity of CRP with all-cause, cardiovascular, and cancer mortality. All statistical tests were performed using SPSS 26.0 and R 4.1.3, where a two-tailed *P* < 0.05 was considered statistically significant.

## Results

### Baseline characteristics

As shown in Table 1, the study included 502 participants with a mean age of 65.18 ± 13.30 years, of whom 369 (73.5%) were male. The higher CRP group had fewer drinkers and a lower prevalence of hypercholesterolemia and use of cholesterol-lowering drugs. They also exhibited higher levels of BMI, triglycerides, uric acid, FPG and HbA1c, but lower levels of HDL-C compared to the lower CRP group (*P* < 0.05).

**Table 1:** Baseline characteristics by C-reactive protein

	Total population	T1	T2	T3	*P* value
*N*	502	168	167	167	
Age, years	65.18 ± 13.30	65.45 ± 13.30	65.94 ± 12.90	64.13 ± 13.70	0.438
Sex, male, *n* (%)	369 (73.50)	132 (78.60)	125 (74.90)	112 (67.10)	0.052
Ideal exercise, *n* (%)					0.110
Yes	107 (21.30)	49 (29.20)	34 (20.40)	24 (14.40)	
No	395 (78.70)	119 (70.80)	133 (79.60)	143 (85.60)	
Smoking status, *n* (%)					0.109
Every day	64 (20.70)	16 (16.20)	19 (18.30)	29 (27.40)	
Some days	18 (5.80)	5 (5.10)	4 (3.80)	9 (8.50)	
Not at all	227 (73.50)	78 (78.80)	81 (77.90)	68 (64.20)	
Drinking, *n* (%)					0.014
Yes	346 (74.40)	123 (79.40)	117 (75.00)	106 (68.80)	
No	119 (25.60)	32 (20.60)	39 (25.00)	48 (31.20)	
Comorbidities, *n* (%)					
Hypertension					0.299
Yes	383 (76.40)	122 (73.10)	127 (76.00)	134 (80.20)	
No	118 (23.60)	45 (26.90)	40 (24.00)	33 (19.80)	
Diabetes					0.282
Yes	139 (27.70)	40 (24.00)	46 (27.50)	53 (31.70)	
No	362 (72.30)	127 (76.00)	121 (72.50)	114 (68.30)	
Hypercholesterolemia					0.016
Yes	274 (61.20)	104 (70.30)	82 (54.70)	88 (58.70)	
No	174 (38.80)	44 (29.70)	68 (45.30)	62 (41.30)	
Cardiovascular disease					0.444
Yes	177 (35.30)	53 (31.70)	64 (38.30)	60 (35.90)	
No	324 (64.70)	114 (68.30)	103 (61.70)	107 (64.10)	
Cancer					0.843
Yes	115 (23.00)	36 (21.40)	39 (23.50)	40 (24.00)	
No	386 (77.00)	132 (78.60)	127 (76.50)	127 (76.00)	
Drugs, *n* (%)					
Hypotensive drugs					0.311
Yes	345 (71.10)	110 (67.50)	114 (70.80)	121 (75.20)	
No	140 (28.90)	53 (32.50)	47 (29.20)	40 (24.80)	
Hypoglycemic drugs					0.359
Yes	126 (25.10)	37 (22.00)	41 (24.60)	48 (28.70)	
No	376 (74.90)	131 (78.00)	126 (75.40)	119 (71.30)	
Cholesterol-lowering drugs					0.047
Yes	198 (47.90)	77 (56.60)	62 (44.00)	59 (43.40)	
No	215 (52.10)	59 (43.40)	79 (56.00)	77 (56.60)	
Body mass index, kg/m^2^	31.82 ± 7.09	29.54 ± 5.47	31.46 ± 6.46	34.54 ± 8.22	< 0.001
Systolic blood pressure, mmHg	131.28 ± 19.24	132.19 ± 19.33	130.63 ± 19.18	131.08 ± 19.32	0.787
Diastolic blood pressure, mmHg	69.30 ± 13.32	70.40 ± 13.54	68.21 ± 13.36	69.22 ± 13.04	0.374
Triglycerides, mmol/L	1.42 (1.12, 2.11)	1.31 (1.10, 1.75)	1.41 (1.03, 2.02)	1.65 (1.17, 2.69)	0.022
Total cholesterol, mmol/L	4.84 ± 1.11	4.69 ± 1.13	4.95 ± 1.11	4.90 ± 1.09	0.086
Low-density lipoprotein cholesterol, mmol/L	2.77 ± 0.90	2.66 ± 0.98	2.94 ± 0.96	2.69 ± 0.70	0.104
High-density lipoprotein cholesterol, mmol/L	1.23 ± 0.38	1.31 ± 0.41	1.22 ± 0.35	1.16 ± 0.35	0.001
Blood urea nitrogen, mmol/L	6.51 ± 3.54	6.04 ± 2.85	6.50 ± 3.31	6.98 ± 4.28	0.054
Creatinine, µmol/L	107.33 ± 69.40	101.80 ± 68.21	104.35 ± 55.07	115.92 ± 81.98	0.141
Uric acid, µmol/L	401.34 ± 110.24	376.18 ± 102.10	405.64 ± 103.43	422.48 ± 120.00	< 0.001
Fasting plasma glucose, mmol/L	6.82 ± 2.35	6.26 ± 1.32	6.57 ± 1.65	7.67 ± 3.37	< 0.001
Hemoglobin A1c, %	6.06 ± 1.06	5.82 ± 0.77	6.02 ± 0.90	6.35 ± 1.35	< 0.001
Mortality, *n* (%)					
All-cause mortality					0.001
Yes	138 (27.50)	30 (17.90)	48 (28.70)	60 (35.90)	
No	364 (72.50)	138 (82.10)	119 (71.30)	107 (64.10)	
Cardiovascular mortality					0.447
Yes	43 (8.60)	12 (7.10)	13 (7.80)	18 (10.80)	
No	459 (91.40)	156 (92.90)	154 (92.20)	149 (89.20)	
Cancer mortality					0.253
Yes	29 (5.80)	6 (3.60)	10 (6.00)	13 (7.80)	
No	473 (94.20)	162 (96.40)	157 (94.00)	154 (92.20)	

T1: ≤ 0.16 mg/dL, T2: 0.16–0.45 mg/dL, T3: > 0.45 mg/dL

### Association of CRP with mortality

**Fig. 2:**
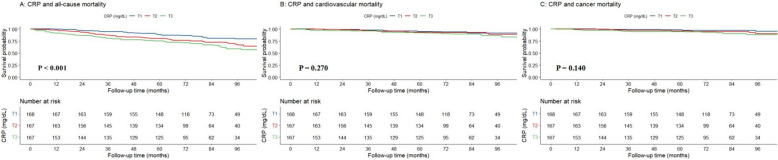
Survival probabilities of all-cause, cardiovascular, and cancer mortality over time by CRP. CRP, C-reactive protein

The Chi-square test showed that the incidence of all-cause mortality increased with increasing levels of CRP (*P* = 0.001), as depicted in Table 1. Kaplan–Meier survival analysis revealed that the change in survival probability, free from mortality events with increasing duration of follow-up, was statistically different among the three CRP groups. The slowest decline in survival probability occurred in the T1 group, and a more rapid decline was observed in the T3 group (*P* < 0.001) as shown in Fig. 2. Multivariate-adjusted Cox regression analysis, presented in Table 2, demonstrated that after adjusting for all confounding variables, individuals with CRP levels in the middle and highest thirds had increased risks of all-cause mortality by 74.2% and 149.7%, respectively (HR 1.742, 95% CI 1.094–2.773, *P* = 0.019; HR 2.497, 95% CI 1.584–3.935, *P* < 0.001). Moreover, the cancer mortality risk of individuals with CRP levels in the highest third also increased by 283.9% (HR 3.839, 95% CI 1.397–10.543, *P* = 0.009). The risk of all-cause and cancer mortality increased by 25.9% and 35.4%, respectively, for each standard deviation increase in CRP (HR 1.259, 95% CI 1.132–1.400, *P* < 0.001; HR 1.354, 95% CI 1.136–1.613, *P* = 0.001).

**Table 2:** Association of C-reactive protein with all-cause, cardiovascular and cancer mortality

	Model 1		Model 2		Model 3	
	HR (95% CI)	*P* value	HR (95% CI)	*P* value	HR (95% CI)	*P* value
All-cause mortality						
T1	Ref.	–	Ref.	–	Ref.	–
T2	1.767 (1.120, 2.789)	0.014	1.886 (1.194, 2.978)	0.007	1.742 (1.094, 2.773)	0.019
T3	2.359 (1.522, 3.658)	< 0.001	2.821 (1.816, 4.381)	< 0.001	2.497 (1.584, 3.935)	< 0.001
*P* for trend	–	< 0.001	–	< 0.001	–	< 0.001
Per 1 SD increment	1.327 (1.190, 1.478)	< 0.001	1.302 (1.178, 1.439)	< 0.001	1.259 (1.132, 1.400)	< 0.001
Cardiovascular mortality						
T1	Ref.	–	Ref.	–	Ref.	–
T2	1.203 (0.549, 2.637)	0.645	1.240 (0.564, 2.725)	0.593	1.288 (0.562, 2.952)	0.550
T3	1.770 (0.852, 3.675)	0.126	1.984 (0.945, 4.163)	0.070	1.974 (0.887, 4.394)	0.096
* P* for trend	–	0.277	–	0.163	–	0.222
Per 1 SD increment	1.274 (1.000, 1.622)	0.050	1.270 (1.012, 1.594)	0.039	1.179 (0.917, 1.516)	0.200
Cancer mortality						
T1	Ref.	–	Ref.	–	Ref.	–
T2	1.838 (0.668, 5.058)	0.239	1.959 (0.710, 5.407)	0.194	1.919 (0.675, 5.454)	0.221
T3	2.584 (0.981, 6.802)	0.055	3.090 (1.161, 8.224)	0.024	3.839 (1.397, 10.543)	0.009
*P* for trend	–	0.157	–	0.076	–	0.217
Per 1 SD increment	1.452 (1.214, 1.736)	< 0.001	1.418 (1.194, 1.684)	< 0.001	1.354 (1.136, 1.613)	0.001

Model 1: unadjusted; Model 2: adjusted for age and sex; Model 3: adjusted for age, sex, ideal exercise, diabetes, cardiovascular disease, cancer, hypoglycemic drugs, body mass index, systolic blood pressure, diastolic blood pressure, total cholesterol, blood urea nitrogen and creatinine

SD, standard deviation; HR, hazard ratio; CI, confidence interval

**Fig. 3:**
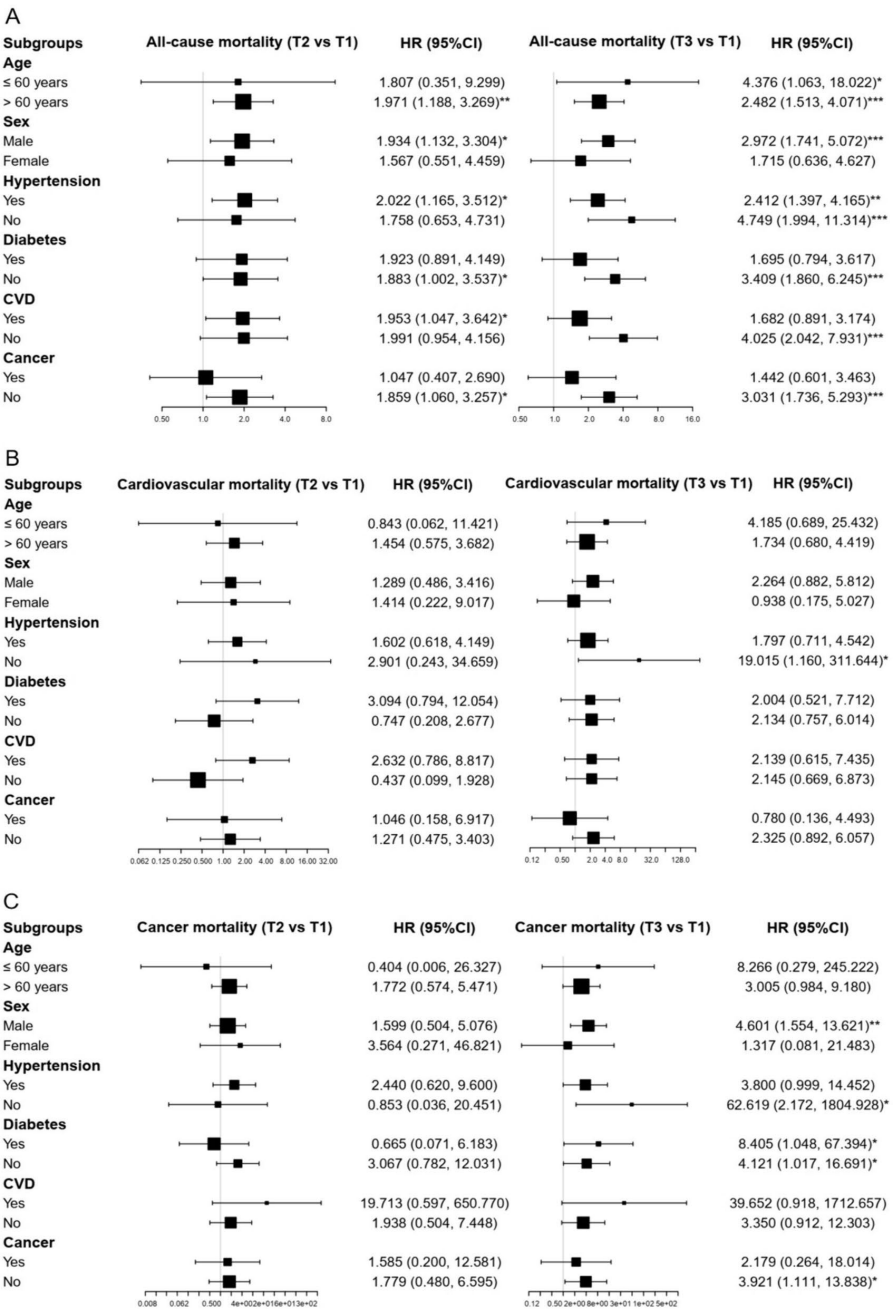
Subgroup analysis forest plot for the association between C-reactive protein and mortality

**Fig. 4:**
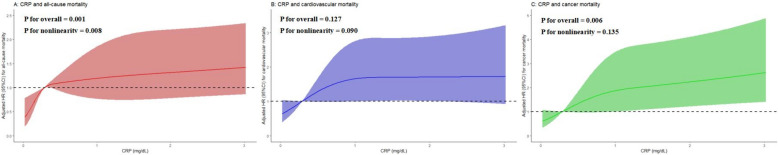
RCS plots for association between CRP with all-cause, cardiovascular, and cancer mortality. RCS, restricted cubic spline; CRP, C-reactive protein; HR, hazard ratio; CI, confidence interval

Subgroup analysis, shown in Table 3 and Fig. 3, indicated that the association between CRP and all-cause mortality remained significant in subgroups of ≤ 60 years, > 60 years, males, hypertension, non-hypertension, non-diabetes, CVD, non-CVD, and non-cancer. The association with cancer mortality was also significant in subgroups of males, non-hypertension, diabetes, non-diabetes, and non-cancer. However, the association with cardiovascular mortality was significant only in the non-hypertension subgroup (*P* < 0.05). As depicted in Fig. 4, the nonlinear association of CRP with all-cause mortality and the linear association with cancer mortality were also confirmed by the RCS analysis (*P* for nonlinearity = 0.008 and 0.135).

**Table 3:** Subgroups analysis for association of CRP with all-cause, cardiovascular and cancer mortality

	All-cause mortality		Cardiovascular mortality		Cancer mortality	
	T2 vs T1	T3 vs T1	T2 vs T1	T3 vs T1	T2 vs T1	T3 vs T1
	HR (95% CI)	HR (95% CI)	HR (95% CI)	HR (95% CI)	HR (95% CI)	HR (95% CI)
Age						
≤ 60 years	1.807 (0.351, 9.299)	4.376 (1.063, 18.022)*	0.843 (0.062, 11.421)	4.185 (0.689, 25.432)	0.404 (0.006, 26.327)	8.266 (0.279, 245.222)
> 60 years	1.971 (1.188, 3.269)**	2.482 (1.513, 4.071)***	1.454 (0.575, 3.682)	1.734 (0.680, 4.419)	1.772 (0.574, 5.471)	3.005 (0.984, 9.180)
Sex						
Male	1.934 (1.132, 3.304)*	2.972 (1.741, 5.072)***	1.289 (0.486, 3.416)	2.264 (0.882, 5.812)	1.599 (0.504, 5.076)	4.601 (1.554, 13.621)**
Female	1.567 (0.551, 4.459)	1.715 (0.636, 4.627)	1.414 (0.222, 9.017)	0.938 (0.175, 5.027)	3.564 (0.271, 46.821)	1.317 (0.081, 21.483)
Hypertension						
Yes	2.022 (1.165, 3.512)*	2.412 (1.397, 4.165)**	1.602 (0.618, 4.149)	1.797 (0.711, 4.542)	2.440 (0.620, 9.600)	3.800 (0.999, 14.452)
No	1.758 (0.653, 4.731)	4.749 (1.994, 11.314)***	2.901 (0.243, 34.659)	19.015 (1.160, 311.644)*	0.853 (0.036, 20.451)	62.619 (2.172, 1804.928)*
Diabetes						
Yes	1.923 (0.891, 4.149)	1.695 (0.794, 3.617)	3.094 (0.794, 12.054)	2.004 (0.521, 7.712)	0.665 (0.071, 6.183)	8.405 (1.048, 67.394)*
No	1.883 (1.002, 3.537)*	3.409 (1.860, 6.245)***	0.747 (0.208, 2.677)	2.134 (0.757, 6.014)	3.067 (0.782, 12.031)	4.121 (1.017, 16.691)*
CVD						
Yes	1.953 (1.047, 3.642)*	1.682 (0.891, 3.174)	2.632 (0.786, 8.817)	2.139 (0.615, 7.435)	19.713 (0.597, 650.770)	39.652 (0.918, 1712.657)
No	1.991 (0.954, 4.156)	4.025 (2.042, 7.931)***	0.437 (0.099, 1.928)	2.145 (0.669, 6.873)	1.938 (0.504, 7.448)	3.350 (0.912, 12.303)
Cancer						
Yes	1.047 (0.407, 2.690)	1.442 (0.601, 3.463)	1.046 (0.158, 6.917)	0.780 (0.136, 4.493)	1.585 (0.200, 12.581)	2.179 (0.264, 18.014)
No	1.859 (1.060, 3.257)*	3.031 (1.736, 5.293)***	1.271 (0.475, 3.403)	2.325 (0.892, 6.057)	1.779 (0.480, 6.595)	3.921 (1.111, 13.838)*

CRP, C-reactive protein; CVD, cardiovascular disease; HR, hazard ratio; CI, confidence interval

*P < 0.05, **P < 0.01, ***P < 0.001

## Discussion

In this real-world longitudinal cohort study, we discovered not only a correlation between CRP levels and all-cause and cancer mortality in gout patients but also identified stratified relationships with all-cause, cardiovascular, and cancer mortality. Furthermore, we confirmed a nonlinear association with all-cause mortality and a linear association with cancer mortality. These findings highlight the risk posed by high CRP levels in gout patients and suggest the significant potential of incorporating CRP into their prognostic management.

CRP, as one of the most common acute-phase proteins, has been proven to be a biomarker for both acute and chronic infections. Its predictive value for the risk and prognosis of various non-infectious diseases, such as rheumatic diseases, metabolism-related diseases, and CVD, has been widely reported [24–26]. For example, a study on the elderly found that high levels of CRP are associated with an increased risk of cardiovascular mortality [27]. In addition, CRP is closely related to the risk of all-cause and cancer mortality in patients with diabetes [28]. Another study showed that CRP can independently predict the risk of all-cause and cardiovascular mortality in patients with acute myocardial infarction [29]. A systematic review and meta-analysis also confirmed the independent association of CRP with all-cause and cardiovascular mortality in patients with diabetes [30]. Furthermore, CRP is closely associated with the mortality rates in patients with heart failure, those undergoing dialysis, or those undergoing transcatheter aortic valve replacement [31–33]. However, despite the above data revealing the impact of high levels of CRP on the mortality of some diseases, its importance in the prognosis management of patients with gout still requires further discussion.

Gout, a chronic metabolic disease, has a globally increasing prevalence, exacerbated by Westernized diets and diverse lifestyles [1, 9]. It is linked to severe pain, hypertension, diabetes, and CVD, raising the risk of all-cause mortality [7, 8, 34–36]. CRP, a nonspecific marker of inflammation, has become a focus in gout studies, with elevated CRP levels indicating inflammation and being directly related to acute gout attacks [37]. High CRP levels are associated with increased cardiovascular risks and lower survival rates, making CRP a critical factor in prognosis management [15, 18]. However, the relationship between CRP levels and mortality risk in gout patients remains unclear. Utilizing the NHANES database, we verified CRP’s correlation with mortality risk in gout patients, revealing stratified associations with all-cause, cardiovascular, and cancer mortality, and a nonlinear association with all-cause mortality. This underscores the importance of monitoring CRP levels for risk management, particularly for cancer mortality, emphasizing the need for vigilance and consideration of CRP’s risk threshold and saturation effect.

In addition, the mechanisms mediating the correlation between CRP and mortality require further exploration. There is evidence suggesting that the activation and release of NLRP3, IL-1β and other pro-inflammatory cytokines play a crucial role in the acute exacerbation of gout. However, it is unclear whether CRP is also involved [38]. Previous studies have indicated that CRP, as a biomarker of systemic inflammation, may reflect a state of chronic infection at high concentrations, thus indirectly increasing the risk of mortality through chronic infection [13]. Furthermore, evidence suggests that CRP can indirectly promote atherosclerosis, thrombosis, and inflammatory stress through mechanisms such as monocyte tissue factor activation, complement activation, leukocyte adhesion, and monocyte recruitment. These processes can lead to CVD and thrombotic events, ultimately increasing the risk of long-term mortality [39–41].

In addition, while CRP levels in patients with gout may relate to acute flare-ups and chronic inflammation, their performance and predictive value for mortality risk could differ in non-gout populations. If this study were conducted among non-gout populations, the results might vary. First, elevated CRP levels in non-gout populations could be associated with other inflammatory diseases, infections, or health conditions, rather than gout specifically. Hence, the relationship between CRP and mortality risk might reflect the impact of these other conditions, not gout itself. Second, due to hyperuricemia and repeated acute inflammatory responses, gout patients may face specific health risks associated with elevated CRP levels, such as CVD. In non-gout populations, the connection between CRP and specific health risks might not be as direct, requiring consideration of other potential mediating factors. Therefore, even though CRP is a nonspecific marker of inflammation detectable across different populations, its predictive value for all-cause and cause-specific mortality risks may be influenced by population characteristics, accompanying health conditions, and other lifestyle factors. Thus, exploring the role and significance of CRP in non-gout populations necessitates further discussion and research to clarify its predictive capabilities and clinical application value in different health contexts.

In addition, whether the increase in mortality is solely due to elevated levels of CRP, rather than underlying comorbidities, is a question that requires further discussion. Generally, an increase in CRP levels could directly indicate underlying inflammatory activity, which is associated with various health risks, including cardiovascular and kidney diseases, all of which could lead to an increase in mortality. However, the role of potential comorbidities cannot be entirely ruled out. Patients with gout may have various comorbidities, such as hypertension, diabetes, and CVD, which are significant risk factors for mortality in themselves. The elevation in CRP levels could result from inflammatory activity from these comorbidities, as well as from gout-related inflammation. Therefore, the increase in mortality could be related both to elevated CRP levels and to underlying comorbidities. The elevation in CRP levels might reflect the state of inflammation in patients with gout, which is directly related to various comorbidities and health risks. These factors together could contribute to an increase in mortality. Thus, determining the exact relationship between elevated CRP levels and increased mortality requires consideration of inflammation, comorbidities, and other potential influencing factors, necessitating further detailed research in the future.

In addition, this study had several limitations. First, the diagnosis of gout in these patients was assessed through a family interview questionnaire, which did not allow for the determination of gout severity; hence, the effect of CRP on mortality could have been mediated by the severity of gout. Second, since this was an observational study, the causal relationship between CRP and the risk of mortality could not be established. Third, this study only analyzed gout participants without adjusting for weight, therefore, the results are limited to this group and do not represent the entire U.S. gout population. Finally, although a large number of covariates were included in this study, it still might have missed some important influencing factors, such as environmental and dietary factors, which are closely related to the quality of life.

## Conclusion

In this longitudinal cohort study, we have demonstrated the association between CRP levels and all-cause, cardiovascular, and cancer mortality in a gouty population. This not only enriches the research on CRP an inflammatory marker in non-inflammatory diseases but also suggests that early detection and intervention of excessive CRP levels in gout patients could significantly reduce premature and excess mortality.

## Data Availability

All raw data included in this study are available on the NHANES website.
